# The Benefits of Early versus Late Therapeutic Intervention in Fabry Disease

**DOI:** 10.1155/2022/3208810

**Published:** 2022-12-30

**Authors:** Mónica Furlano, Elisabet Ars, Anna Matamala, Vicens Brossa, Joan Martí, Maria del Prado-Venegas, Jaume Crespi, Esther Roe, Roser Torra

**Affiliations:** ^1^Inherited Kidney Diseases, Nephrology Department, Fundació Puigvert, Instituto de Investigaciones Biomédicas Sant Pau (IIB-Sant Pau), Medicine Department-Universitat Autónoma de Barcelona, Barcelona, Spain; ^2^Molecular Biology Laboratory, Fundació Puigvert, Instituto de Investigaciones Biomédicas Sant Pau (IIB Sant Pau), Barcelona, Spain; ^3^Inherited Kidney Diseases, Nursing Department, Fundació Puigvert, Barcelona, Spain; ^4^Cardiology Department, Hospital de la Santa Creu i Sant Pau, Barcelona, Spain; ^5^Department of Neurology (Stroke Unit), Hospital de la Santa Creu i Sant Pau, Barcelona, Spain; ^6^Department of Otorhinolaryngology, Hospital de la Sta Creu i Sant Pau, Barcelona, Spain; ^7^Ophtalmology Department, Hospital de Sant Pau-Universitat Autonoma Barcelona, Barcelona, Spain; ^8^Dermatology Department, Hospital de la Santa Creu i Sant Pau, Barcelona, Spain

## Abstract

**Background:**

Fabry disease (FD) is an X-linked lysosomal storage disorder caused by pathogenic variants of the *GLA* gene. Heterozygous female patients may show much more variability in clinical manifestations, ranging from asymptomatic to full-blown disease. Because of this heterogeneous clinical picture in women, the diagnosis of FD has typically been delayed for more than a decade, and the optimal time to initiate treatment remains controversial. *Case Presentation*. Here, we present two unrelated female patients diagnosed with FD harbouring the same pathogenic *GLA* variant. We discuss the implications of initiating specific therapy at different stages of the disease, with and without organ involvement (early versus late therapeutic intervention).

**Conclusions:**

These clinical cases suggest that initiating specific treatment at an earlier age in women with FD may prevent organ involvement and associated clinical events.

## 1. Introduction

Fabry disease (FD) is a lysosomal storage disorder with X-linked transmission caused by pathogenic variants in the *GLA* gene encoding *α*-galactosidase A (*α*-Gal A). Deficiency or decreased activity of this enzyme results in accumulation of its main unmetabolized substrate (globotriaosylceramide [GL-3]) and its derivatives (e.g., lyso-Gb3) in various tissues and organs [1–3].

The disease can be divided into a severe, classical phenotype clinically characterised by skin lesions (angiokeratoma), recurrent burning pain (acroparaesthesias), cornea verticillata, hypohidrosis, cardiac and renal injury, and cerebral ischaemia, and a generally milder nonclassical phenotype, also referred to as late-onset FD, characterised by a more variable disease course [4, 5]. Heterozygous females have variable clinical manifestations, depending on the inactivation pattern of the *X* chromosome, ranging from asymptomatic to full-blown disease [6]. *α*-Gal A levels in females may be within the normal range, so FD diagnosis relies on the sequencing of the *GLA* gene [7].

Migalastat was approved in 2016. It is an oral pharmacological chaperone, which stabilises *α*‐Gal A when it has an amenable mutation (an estimated 35–50% of patients with FD overall), increasing enzyme trafficking to lysosomes and thus intracellular *α*‐Gal A activity [8–10].

There are more than 1,000 different disease-causing variants known in the *GLA* gene to be associated with FD (Human Gene Mutation Database, https://www.hgmd.org/). The occurrence of disease complications is highly age-dependent. Timely diagnosis and early therapeutic intervention are favourable for disease prognosis and can prevent the occurrence of or even partially reverse organ lesions caused by FD [3]. However, much of the published literature describe outcomes after late treatment initiation once substantial organ damage has already occurred.

Here, we report the case of two women with the p. Ile270Thr pathogenic variant who have been treated with migalastat for 11 and 5 years, respectively, and the differences in functional class deterioration depending on early therapeutic intervention.

## 2. Case presentation

### 2.1. Case 1

**Figure 1:**
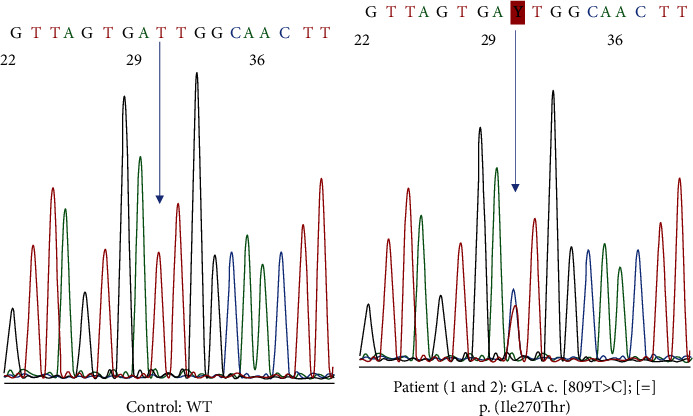
Sanger sequencing of exon 6 of the GLA gene. (a) Normal control showing wild-type (WT) sequence. (b) Patient sequence with GLA variant c.809T >C p.(Ile207Thr) in heterozygosity. The arrow indicates the nucleotide position with the sequence variant.

We present the case of a 38-year-old female with 10 years of follow-up since migalastat initiation. Her previous medical history included gastrointestinal symptoms, including episodes of diarrhoea at 18 years of age. An intestinal biopsy was performed to relate the gastrointestinal symptoms to FD, but no GL-3 deposits were detected. Therefore, the patient was diagnosed with irritable bowel syndrome. She also noticed acroparaesthesia and whorled opacities in the cornea (cornea verticillata). Sequence analysis of the *GLA* gene in the patient, in comparison with the wild-type sequence (Figure 1), revealed a single nucleotide point mutation in heterozygosis at nucleotide c.809T >C p.(Ile270Thr) (*GLA* variant: NM_000169.3). This variant results in the change from isoleucine to threonine at position 270 of the exon 6 [11] This variant has not been found in the population database gnomAD, and it is predicted to be pathogenic by most computational predictive programs (BayesDel_addAF, DANN, DEOGEN2, FATHMM-MKL, LIST-S2, M-CAP, MVP, MutationAssessor, MutationTaster, SIFT) as well as by the meta-predictor REVEL (score = 0.97). This variant has been associated with the classic Fabry phenotype, and its amenability to migalastat therapy has been confirmed in the GLP HEK cell-based assay [12]. Taking into account all this evidence and following the American College of Medical Genetics and Genomics guidelines for the interpretation of sequence variants [13], the variant *GLA* c.809T >C p. (Ile270Thr) is pathogenic.

**Figure 2:**
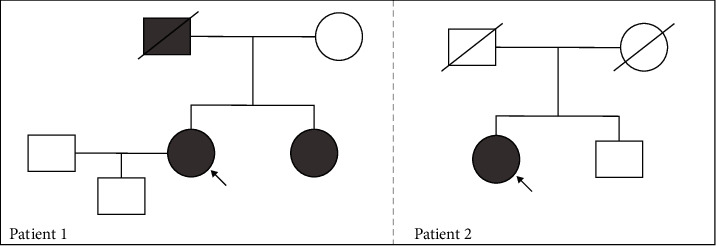
Pedigrees. The proband is indicated with an arrow. Striped boxes represent males with Fabry disease; striped circles represent females with Fabry disease; slash indicates deceased.

Regarding her family history, her father had classical FD and, after undergoing a kidney and heart transplant, finally died at age 42. Her 43-year-old sister was also diagnosed with the disease and remains asymptomatic. Her 13-year-old son was found to be unaffected in a prenatal diagnosis (Figure 2).

**Figure 3:**
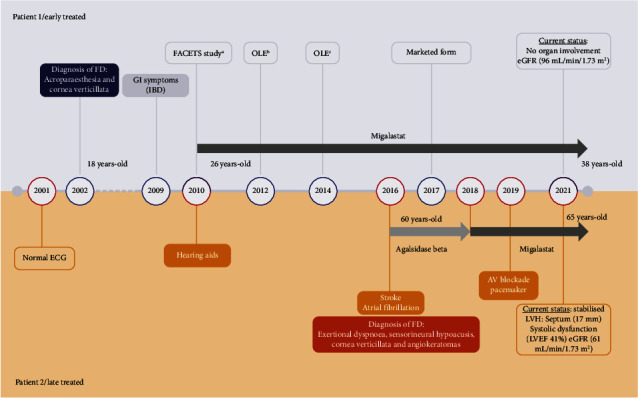
Medical history and occurrence of events over time in two female patients treated at different stages of FD. ^*a*^AT1001-011 (NCT00925301), ^*b*^AT1001-041 (NCT01458119), and ^*c*^AT1001-042 (NCT02194985). AV, atrioventricular; ECG, echocardiogram; eGFR, estimated glomerular filtration rate, normal: >90 and >60 for omen >50 years^15^; FD, Fabry disease; GI, gastrointestinal; IBD, irritable bowel syndrome; LVEF, left ventricular ejection fraction; LVH, left ventricular hypertrophy; OLE, open-label extension.

In October 2010, after confirming eligibility criteria, she was included in a clinical trial to receive migalastat for 24 months (FACETS study [8]) (Figure 3).

**Figure 4:**
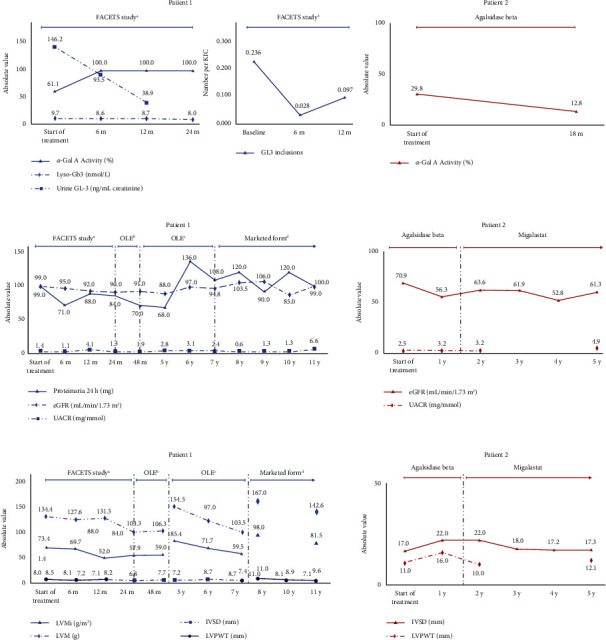
Changes in (a) Metabolism. (b) Renal. (c) Cardiac Parameters with treatment at different stages in two cases of FD. *α*‐Gal A is expressed as a percentage of normal (% normal). Normal white blood cell activity: 22 nmol free 4 MU released/mg protein/h^7^ (patient 1); normal laboratory reference activity on dried blood spots samples: 4.7–18.8 *μ*mol/L/h (patient 2); normal Lyso-Gb3: ≤0.6 nm/L; normal urine GL-3: <33.8 ng/mL; normal protein excretion rate: <150 mg/24 hours; normal eGFR: >90 mL/min/1.73 m^2^; normal UACR: <3 mg/mmol; normal LVMI: 43–95 g/m^2^; normal LVPWT: 6–10 mm; normal IVSD: 6–9 mm. Dotted lines define the end of study (case 1) or treatment switch (case 2). In the cardiac parameters graph of case 1, gaps indicate a different method of measurement. Case 1: urine GL-3 and GL-3 inclusions were measured up to month 12 of the FACETS study; LVMI and LVM not measured at 8, 9, and 10 months; LVPWT measured upto 12 months of the FACETS study; case 2: UACR and LVPWT not measured at years 3 and 4. ^*a*^NCT00925301, ^*b*^AT1001-041; ^*c*^AT1001-042. *α*‐Gal A, alpha-galactosidase A; eGFR, estimated glomerular filtration rate; FD, Fabry disease; GL-3, globotriaosylceramide; IVSD, interventricular septal thickness at end diastole; LVMI, left ventricular mass index; LVPWT, left ventricular posterior wall thickness; Lyso-Gb3, globotriaosylsphingosine; OLE, open-label extension; UACR, urine albumin-to-creatinine ratio.

Her glomerular filtration rate (GFR) at baseline, measured by plasma clearance of iohexol, was normal, and her GL-3 in urine was >4 times the upper limit of normal, fulfilling the criteria for the clinical trial. At enrolment, the number of GL-3 inclusions in interstitial capillary cells (KIC GL-3) obtained from a kidney biopsy sample was 0.236. Likewise, GL-3 inclusions were documented in podocytes endothelial cells (data not shown). *α*-Gal A activity in white blood cells was decreased by almost 40% [8] and plasma lyso-Gb3 was increased (Figure 4(a)). The protein excretion rate was within the normal reference values (Figure 4(b)). The echocardiographic evaluation confirmed preserved dimension and function (Figure 4(c)).

The patient's pain (Brief Inventory Pain (BPI)) [14] and gastrointestinal symptoms (Gastrointestinal Symptom Rating Scale (GSRS)) [15] remained stable throughout the follow-up.

After 6 months, migalastat treatment reduced KIC GL-3 inclusions, urine GL-3, and plasma lyso-Gb3. Importantly, enzyme activity was fully restored (Figure 4(a)). Renal and cardiac parameters were kept at physiological values (Figures 4(b) and 4(c)). Six months later (12 months after migalastat initiation), plasma lyso-Gb3 levels remained stable without further reduction, while urine GL-3 concentration showed a meaningful decrease. In addition, *α*‐Gal A activity remained stable. Renal biopsy revealed a slight increase in KIC GL-3 inclusions (Figure 4(a)), but overall renal function remained normal (Figure 4(b)). Notably, a decrease in the left ventricular mass index and in left ventricular posterior wall thickness parameters were observed (Figure 4(c)). At the end of the trial (24 months after migalastat initiation), the patient remained stable, with unremarkable changes (Figures 4(a)–4(c)).

In 2012, after completing the pivotal study, the patient was enrolled in the open-label extension (OLE) study (AT1001-041) to receive migalastat for an additional 24 months (Figure 3). Forty-eight months (4 years) after migalastat initiation, all scores related to renal and cardiac parameters fell within normal reference values (Figures 4(b) and 4(c)).

In 2014, at age 30. The patient was included in the second extension of the study (AT1001-042), where she received migalastat for another 36 months (Figure 3). At the end of this period (7 years on migalastat treatment), renal and cardiac values were still within physiological ranges (Figures 4(b) and 4(c)).

Since 2017, our patient has continued treatment with migalastat in its marketed form. Currently, she remains stable, with no significant albuminuria and no organ involvement (Figures 3, 4(b), and 4(c)).

### 2.2. Case 2

We present the case of a 65-year-old woman diagnosed with FD and severe organ involvement treated with migalastat for the last 3 years. The patient had a long medical history, including multiple conditions such as cardiac hypertrophy with preserved left ventricular ejection fraction (LVEF) and diastolic dysfunction, exertional dyspnoea, cutaneous lupus disease, primary antiphospholipid syndrome, dyslipidaemia, hypertension, and atrophic gastritis. She is currently being treated with torasemide, amiodarone, enalapril, bisoprolol, acenocoumarol, and pravastatin. In 2016, she suffered a stroke of the right middle cerebral artery along with paroxysmal atrial fibrillation. At that time, typical FD manifestations (severe sensorineural hypoacusis, cornea verticillata, and angiokeratomas) were also detected (Figure 3). Genetic testing demonstrated the presence of a pathogenic sequence variant in the *GLA* gene: c.809T >C p.(Ile270Thr), the same as the previous case although not a relative (Figure 1). Plasma *α*‐Gal A activity was decreased (less than 30%) (Figure 4(a)). Cardiac hypertrophy was then attributed to FD. Fabry-specific therapy was immediately initiated with enzyme replacement therapy (ERT) (agalsidase beta) (Figure 3). Regarding her family history, although her mother had a hypertrophic cardiomyopathy and her father and brother had ischaemic heart disease and sensorineural hearing loss, genetic testing was negative for FD (Figure 2).

In 2018, the patient requested to be switched from ERT to migalastat. During follow-up, the patient experienced unexplained syncopes and conduction disturbances (first-degree auriculoventricular blockade with right ventricle bundle branch block and left anterior hemiblock), which were confirmed by a pathological electrophysiological study showing a prolonged Hiss-Ventricular (HV) interval, and the patient was fitted with a permanent dual chamber pacemaker in 2019 (Figure 3).

Currently, the patient continues on migalastat and is stabilised with moderate systolic dysfunction (LVEF 41%), an IVSD of 17 mm, a preserved, stable eGFR of 61 mL/min/1.73 m^2^ (Figures 3, 4(b), and 4(c)) and no microalbuminuria.

## 3. Discussion

This report describes two unrelated female patients with FD harbouring the same *GLA* p. Ile270Thr pathogenic variant and the effect of early treatment, not only on symptoms and surrogates, including LVM and GFR, but also on clinical events. The literature has extensively described this variant in association with the classic Fabry phenotype [11, 16–21]. Specific features of advanced disease, such as cardiac fibrosis or severe renal dysfunction, are hallmarks of irreversible organ damage. Thus, it is mandatory to start treatment before the occurrence of irreversible manifestations to achieve better outcomes [22–24]. This is in line with the two cases described here. In the first case, treatment was initiated at an early stage of the disease, before renal or cardiac involvement, while in the second case, treatment was started at a late stage, with severe and irreversible cardiac involvement.

As previously mentioned, the patient in the first case initiated FD-specific therapy upon inclusion in the phase III FACETS study at the age of 26 years, before any evident organ damage. After 6 months of migalastat treatment, KIC GL-3 inclusions, urine GL-3, and plasma lyso-Gb3 were reduced, and *α*-Gal A activity was fully restored. These results are consistent with recent findings from the migalastat phase III ATTRACT study [8, 25]. Moreover, the patient remained asymptomatic with no organ involvement for the whole follow-up. This is doubly remarkable when talking about a variant associated with a classic phenotype, given that these females are more likely to develop complications than nonclassical ones [5]. The second patient was diagnosed late in the course of the disease, when cardiac hypertrophy was already severe. Although she started ERT immediately upon diagnosis and was later switched to migalastat, her cardiac hypertrophy did not improve, and she even required a pacemaker. The patient's many comorbidities may have also played a role in her poorer outcome. However, she has not developed Fabry nephropathy, as although her eGFR is not optimal, she shows no microalbuminuria, a hallmark of the disease. This may be an example of how FD therapy does not reverse organ involvement but may prevent damage to other organs, such as the kidney.

Even so, it cannot be ruled out that the different disease severity in the two cases stems from a different *X* inactivation pattern. According to previous studies, preferential inactivation of the wild-type *GLA* allele is associated with an early onset of disease and rapid progression, compared to a milder disease when the mutated allele is preferentially inactivated [26, 27]. Conversely, other studies [28, 29] reported that *X* inactivation patterns in symptomatic females did not differ from those of female controls of the same age. Some of these discrepancies may arise from the fact that disease progression is also related to a cross-correction mechanism that degrades over time, explaining why women with FD develop more symptoms with age [28]. This highlights the need for a larger series to support our findings.

In conclusion, these clinical cases at different stages of disease progression highlight the importance of early diagnosis and timely treatment initiation in patients with FD and provide further evidence of the long-term efficacy and safety of migalastat in patients with amenable GLA mutations.

## Data Availability

The datasets during and/or analysed during the current study are available from the corresponding author upon request.
